# Supplementary material for: The Influence of Heteroresistance on Minimum Inhibitory Concentration, Investigated Using Weak-Acid Stress in Food Spoilage Yeasts
Source: Appl Environ Microbiol. 2023 May 31;89(6):e00125-23. doi: 10.1128/aem.00125-23 (PMC10304792; doi:10.1128/aem.00125-23)
Supplement: Supplemental file 1 — Supplemental method, Tables S1 to S4, and Fig. S1 to S6. Download aem.00125-23-s0001.docx, DOCX file, 1.2 MB [file aem.00125-23-s0001.docx]

**Supplementary Materials**

**The influence of heteroresistance on minimum inhibitory concentration (MIC), investigated using weak-acid stress in food spoilage yeasts**

Joseph Violet^1^, Joost Smid^2^, Annemarie Pielaat^2^, Jan-Willem Sanders^2^ and Simon V. Avery^1,^*

^1^School of Life Sciences, University of Nottingham, Nottingham NG7 2RD, United Kingdom

^2^Unilever Foods Innovation Centre, Bronland 14, 6708 WH Wageningen, the Netherlands

**Supplementary Method**

Determination of cell density effect

Exponential cultures of *Z. parabailii* were suspended in fresh YEPD broth, pH 4. Appropriate dilutions of cell suspension were aliquoted across 96-well flat bottomed microtiter plates (Starlab) and supplemented with sorbic acid to final concentrations of 5.7 and 5.65mM for strains 7445 and 679 respectively, in final volumes of 150 µl per well and either final OD_600_ 0.01 (~10,000 cells/well) or OD_600_ 0.0001 (~100 cells/well). Plates were taped, bagged and incubated at 23°C for 21 days. Wells with visible growth were enumerated and converted to experimental growth probability [(number of wells with visible growth) / (total number of wells)] at the two inoculum sizes. Equation S1 was employed to convert experimental growth probability at 100 cells/well to a predicted growth probability at 10,000 cells/well, based on the increased probability of a resistant cell being present in the larger inoculum. The proportional difference between this predicted growth probability and the experimental growth probability at 10,000 cells/well (from the experiment described above) was calculated using Equation S2 and termed ‘Cell-density effect (% increase in probability)’. This ‘Cell density effect’ gives the proportion of the growth probability at 10,000 cells/well which is not accountable for by the increased probability of a resistant cell being present (i.e. the probability increase expected purely from the increased cell density between 100 and 10,000 cells/well).

*Equation S1*

$P\left( growth in at least 1 of 100 wells, each containing 100 cells \right)= 1-{(1-P\left( growth in 1 well containing 100 cells \right))}^{100}$

*Equation S2*

$$y=100*\frac{\begin{aligned} P\left( growth in 1 well containing 10,000 cells \right)- \\ P\left( growth in at least 1 of 100 wells, each containing 100 cells \right) \end{aligned}}{P(growth in one well containing 10,000 cells)}$$

$y$ = Cell density effect (%)

**Supplementary Tables**

**Table S1: Initial panel of *Zygosaccharomyces* strains**

| Strain reference no. | Alias ID(s) | Source of Isolation | Previous designation | New Designation | Genome-sequence sample number (ENA)^a^ |
| --- | --- | --- | --- | --- | --- |
| 671 | CIMSCEE 05 |  | *Zygosaccharomyces bailii* | *Zygosaccharomyces pseudobailii* | SAMEA112469141 |
| 838 | CIMSCEE 639 |  | *Zygosaccharomyces bailii* | *Zygosaccharomyces pseudobailii* | SAMEA112469142 |
| 947 |  | Canned fruit, USA | *Zygosaccharomyces bailii* | *Zygosaccharomyces parabailii* | SAMEA112469143 |
| 1474 |  | Sauce, Italy | *Zygosaccharomyces bailii* | *Zygosaccharomyces parabailii* | SAMEA112469144 |
| 1727 |  | Vinaigrette | *Zygosaccharomyces bailii* | *Zygosaccharomyces parabailii* | SAMEA112469145 |
| 1730 |  | Ketchup | *Zygosaccharomyces pseudobailii* | *Zygosaccharomyces pseudobailii* | SAMEA112469146 |
| 3112 |  |  | *Zygosaccharomyces bailii* | *Zygosaccharomyces parabailii* | SAMEA112469147 |
| 3492 |  | Ketchup, Russia | *Zygosaccharomyces bailii* | *Zygosaccharomyces parabailii* | SAMEA112469148 |
| 3500 |  | Kombucha | *Zygosaccharomyces bailii* | *Zygosaccharomyces bailii* | SAMEA112469149 |
| 3696 |  | Onion | *Zygosaccharomyces bailii* | *Zygosaccharomyces parabailii* | SAMEA112469150 |
| 3697 |  | low fat tartare sauce | *Zygosaccharomyces pseudobailii* | *Zygosaccharomyces pseudobailii* | SAMEA112469151 |
| 3698 |  | Chow chow | *Zygosaccharomyces bailii* | *Zygosaccharomyces parabailii* | SAMEA112469152 |
| 3699 |  | Creamy caesar dressing | *Zygosaccharomyces parabailii* | *Zygosaccharomyces parabailii* | SAMEA112469153 |
| 3700 |  | Pickles | *Zygosaccharomyces bailii* | *Zygosaccharomyces pseudobailii* | SAMEA112469154 |
| 3703 |  | vinaigrette olive/citron | *Zygosaccharomyces parabailii* | *Zygosaccharomyces parabailii* | SAMEA112469155 |
| 3704 |  | Ketchup, Russia | *Zygosaccharomyces bailii* | *Zygosaccharomyces parabailii* | SAMEA112469156 |
| 3705 |  | Ketchup curry, Czech republic | *Zygosaccharomyces parabailii* | *Zygosaccharomyces parabailii* | SAMEA112469157 |
| 3914 | 119^b,c,d^ | Netherlands | *Zygosaccharomyces bailii* | *Zygosaccharomyces parabailii* | SAMEA112469158 |
| 3918 | NCYC 2932, HS 107 | Ketchup, UK | *Zygosaccharomyces bailii* | *Zygosaccharomyces parabailii* | SAMEA112469159 |
| 3942 |  | Yoghurt mayonnaise | *Zygosaccharomyces bailii* | *Zygosaccharomyces parabailii* | SAMEA112469160 |
| 3959 |  | Ice Tea concentrate | *Zygosaccharomyces parabailii* | *Zygosaccharomyces parabailii* | SAMEA112469161 |
| 4028 | CMCC 3474, NCYC 1766 | Black currant & grape juice, UK | *Zygosaccharomyces bailii* | *Zygosaccharomyces parabailii* | SAMEA112469162 |
| 4240 |  | 1000 Islands dressing | *Zygosaccharomyces bailii* | *Zygosaccharomyces parabailii* | SAMEA112469163 |
| 4391 | 4^b,c,d^, CFT -1 | canned tea, USA | *Zygosaccharomyces bailii* | *Zygosaccharomyces parabailii* | SAMEA112469164 |
| 4392 | 108^b,c,d^ | Tomato based product, UK | *Zygosaccharomyces bailii* | *Zygosaccharomyces parabailii* | SAMEA112469165 |
| 4411 | KL 1522, 106^b,c,d^ | Tomato based product, UK | *Zygosaccharomyces bailii* | *Zygosaccharomyces parabailii* | SAMEA112469166 |
| 4847 |  | Herring in tomato sauce | *Zygosaccharomyces bailii* | *Zygosaccharomyces parabailii* | SAMEA112469167 |
| 7406 |  | Ice tea production environment, Nigeria | *Zygosaccharomyces bailii* | *Zygosaccharomyces parabailii* | SAMEA112469168 |
| 7445 |  | Ice tea production, Mexico | *Zygosaccharomyces parabailii* | *Zygosaccharomyces parabailii* | SAMEA112469169 |
| 7472 |  | Mayonnaise, Netherlands | *Zygosaccharomyces pseudobailii* | *Zygosaccharomyces pseudobailii* | SAMEA112469170 |
| 7479 |  | Mayonnaise, Philippines | *Zygosaccharomyces parabailii* | *Zygosaccharomyces parabailii* | SAMEA112469171 |
| 7750 |  | Spoiled Vinaigrette, western Europe | *Zygosaccharomyces bailii* | *Zygosaccharomyces parabailii* | SAMEA112469172 |
| 7756 |  | Ice Tea concentrate, Germany / Portugal | *Zygosaccharomyces bailii* | *Zygosaccharomyces bailii* | SAMEA112469173 |
| 7768 |  | Ice Tea concentrate, Portugal | *Zygosaccharomyces bailii* | *Zygosaccharomyces bailii* | SAMEA112469174 |
| 7769 |  | Tartare Sauce, Poland / Czech republic | *Zygosaccharomyces pseudobailii* | *Zygosaccharomyces pseudobailii* | SAMEA112469175 |
| 7770 |  | Concentrate dispensing system, Portugal | *Zygosaccharomyces bailii* | *Zygosaccharomyces bailii* | SAMEA112469176 |
| 7771 |  | Concentrate dispensing system, Portugal | *Zygosaccharomyces bailii* | *Zygosaccharomyces bailii* | SAMEA112469177 |
| 7773 | CECT11931 | Juice, lees of pear must ,Unknown | *Zygosaccharomyces bailii* | *Zygosaccharomyces bailii* | SAMEA112469178 |
| 7774 | MUCL 28823 | Apple juice , Netherlands | *Zygosaccharomyces bailii* | *Zygosaccharomyces parabailii* | SAMEA112469179 |
| 7776 | DBVPG 6451 |  | *Zygosaccharomyces bailii* | *Zygosaccharomyces pseudobailii* | SAMEA112469180 |
| 7777 | DBVPG 6378 | Mayonnaise, Netherlands | *Zygosaccharomyces bailii* | *Zygosaccharomyces parabailii* | SAMEA112469181 |
| 7780 |  |  | *Zygosaccharomyces bailii* | *Zygosaccharomyces parabailii* | SAMEA112469182 |
| 7781 | DBVPG 6454 | Netherlands | *Zygosaccharomyces bailii* | *Zygosaccharomyces parabailii* | SAMEA112469183 |
| 7784 | DBVPG 6456 | Juice, Italy | *Zygosaccharomyces bailii* | *Zygosaccharomyces bailii* | SAMEA112469184 |
| 7788 | CECT11922 | Orange-juice concentrate, Brazil | *Zygosaccharomyces bailii* | *Zygosaccharomyces bailii* | SAMEA112469185 |
| 7789 | CCRC 22630 | Honey | *Zygosaccharomyces bailii* | *Zygosaccharomyces parabailii* | SAMEA112469186 |
| 7790 | CIMSCEE 639 |  | *Zygosaccharomyces bailii* | *Zygosaccharomyces pseudobailii* | SAMEA112469187 |
| 7791 | CIMSCEE 05 |  | *Zygosaccharomyces bailii* | *Zygosaccharomyces pseudobailii* | SAMEA112469188 |
| 7794 | BRAS 749 | Poland | *Zygosaccharomyces bailii* | *Zygosaccharomyces bailii* | SAMEA112469189 |
| 7795 |  | orange wine, Zaïre | *Zygosaccharomyces bailii* | *Zygosaccharomyces bailii* | SAMEA112469190 |
| 7796 |  |  | *Zygosaccharomyces parabailii* | *Zygosaccharomyces parabailii* | SAMEA112469191 |
| 7799 |  | fermented tea | *Zygosaccharomyces bailii* | *Zygosaccharomyces bailii* | SAMEA112469192 |
| 7800 |  | fermented tea | *Zygosaccharomyces bailii* | *Zygosaccharomyces bailii* | SAMEA112469193 |
| 7805 |  | sweet wine, Italy | *Zygosaccharomyces bailii* | *Zygosaccharomyces bailii* | SAMEA112469194 |
| 7807 |  | alcoholic fermented beverage | *Zygosaccharomyces bailii* | *Zygosaccharomyces parabailii* | SAMEA112469195 |
| 7808 | ATCC MYA-4549 |  | *Zygosaccharomyces parabailii* | *Zygosaccharomyces parabailii* | SAMEA112469196 |
| 7809 | NCYC 563^c^, 2^a,b^ | Sorghum brandy mash, UK | *Zygosaccharomyces bailii* | *Zygosaccharomyces pseudobailii* | SAMEA112469197 |
| 7810 | 4^a,b,c^, CFT1 | Canned ice tea, USA | *Zygosaccharomyces bailii* | *Zygosaccharomyces parabailii* | SAMEA112469198 |
| 7811 | 5^a,b,c^ CFT-10 | Canned fruit, USA | *Zygosaccharomyces bailii* | *Zygosaccharomyces parabailii* | SAMEA112469199 |
| 7812 | 6^b,c,d^ | Bottled ice tea, USA | *Zygosaccharomyces bailii* | *Zygosaccharomyces parabailii* | SAMEA112469200 |
| 7814 | 8^b,c,d^ | Ice tea, USA | *Zygosaccharomyces bailii* | *Zygosaccharomyces parabailii* | SAMEA112469201 |
| 7815 | 9^b,c,d^ | Carbonated soft drink, USA | *Zygosaccharomyces bailii* | *Zygosaccharomyces parabailii* | SAMEA112469202 |
| 7816 | 10^b,c,d^ | Ice tea, USA | *Zygosaccharomyces bailii* | *Zygosaccharomyces parabailii* | SAMEA112469203 |
| 7817 | 11^b,c,d^ | Ice tea, USA | *Zygosaccharomyces bailii* | *Zygosaccharomyces parabailii* | SAMEA112469204 |
| 7819 | 12^b,c,d^ | Soft drink, USA | *Zygosaccharomyces bailii* | *Zygosaccharomyces parabailii* | SAMEA112469205 |
| 7820 | 15^b,c,d^ CMCC 3299 | Salad dressing, Netherlands | *Zygosaccharomyces bailii* | *Zygosaccharomyces pseudobailii* | SAMEA112469206 |
| 7823 | 18^b,c,d^ NCYC 3410, CMCC 2959 | Herring in tomato sauce, UK | *Zygosaccharomyces bailii* | *Zygosaccharomyces parabailii* | SAMEA112469207 |
| 7824 | 19^b,c,d^ NCYC 3407, CMCC 2589 | Spoiled soft drink (lemon tea), UK | *Zygosaccharomyces bailii* | *Zygosaccharomyces parabailii* | SAMEA112469208 |
| 7825 | 20^b,c,d^ NCYC 3414, CMCC 2968 | Orange juice concentrate, UK | *Zygosaccharomyces bailii* | *Zygosaccharomyces parabailii* | SAMEA112469209 |
| 7826 | 21^b,c,d^ CMCC 2960 | Herring in tomato sauce, UK | *Zygosaccharomyces bailii* | *Zygosaccharomyces parabailii* | SAMEA112469210 |
| 7828 | 80^b,c,d^ CMCC 3229 | Mexican Topping sauce, UK | *Zygosaccharomyces bailii* | *Zygosaccharomyces parabailii* | SAMEA112469211 |
| 7829 | 105^b,c,d^ NCYC 2933, CMCC3630 | Ketchup, UK | *Zygosaccharomyces bailii* | *Zygosaccharomyces parabailii* | SAMEA112469212 |
| 7830 | 106^b,c,d^ | Ketchup, UK | *Zygosaccharomyces bailii* | *Zygosaccharomyces parabailii* | SAMEA112469213 |
| 7831 | 107^b,c,d^ KL 1750, NCYC 2932 | Ketchup, UK | *Zygosaccharomyces bailii* | *Zygosaccharomyces parabailii* | SAMEA112469214 |
| 7832 | 108^b,c,d^ KL 2436, NCYC 2931 | Ketchup, UK | *Zygosaccharomyces bailii* | *Zygosaccharomyces parabailii* | SAMEA112469215 |
| 7833 | 112^b,c,d^ | Ice tea, Belgium | *Zygosaccharomyces bailii* | *Zygosaccharomyces parabailii* | SAMEA112469216 |
| 7834 | 114^b,c,d^ | Ice tea, Belgium | *Zygosaccharomyces bailii* | *Zygosaccharomyces parabailii* | SAMEA112469217 |
| 7835 | 119^b,c,d^ NCYC 2927 | Soft drink, Netherlands | *Zygosaccharomyces bailii* | *Zygosaccharomyces parabailii* | SAMEA112469218 |
| 7836 | 194^b,c,d^ DBVPG 6924 | Honey, China | *Zygosaccharomyces bailii* | *Zygosaccharomyces pseudobailii* | SAMEA112469219 |
| 7837 | 280^b,c,d^ | Ice Tea, South Africa | *Zygosaccharomyces bailii* | *Zygosaccharomyces parabailii* | SAMEA112469220 |
| 7838 | 362^b,c,d^ | Factory isolate, Turkey | *Zygosaccharomyces bailii* | *Zygosaccharomyces parabailii* | SAMEA112469221 |
| 7839 | 475^b,c,d^ | Factory isolate, Brazil | *Zygosaccharomyces bailii* | *Zygosaccharomyces parabailii* | SAMEA112469222 |
| 7840 | 503^b,c,d^ NCYC 3302 | Kombucha, UK | *Zygosaccharomyces bailii* | *Zygosaccharomyces bailii* | SAMEA112469223 |
| 7841 | 505^b,c,d^ NCYC 3307 | Kombucha, UK | *Zygosaccharomyces bailii* | *Zygosaccharomyces bailii* | SAMEA112469224 |
| 7842 | 593^b,c,d^ NCYC 3378 | Syrup room, Soft Drinks factory, Philippines | *Zygosaccharomyces bailii* | *Zygosaccharomyces parabailii* | SAMEA112469225 |
| 7843 | 594^b,c,d^ NCYC 3379 | Factory isolate, Philippines | *Zygosaccharomyces bailii* | *Zygosaccharomyces bailii* | SAMEA112469226 |
| 7845 | 595^b,c,d^ | Dried fruit, Spain | *Zygosaccharomyces bailii* | *Zygosaccharomyces parabailii* | SAMEA112469227 |
| 7846 | NCYC 1416^b,c^, ATCC 58445 | Brewery, Japan | *Zygosaccharomyces bailii* | *Zygosaccharomyces bailii* | SAMEA112469228 |
| 7847 | NCYC 1766^b,c,d^ | Grape and blackcurrant juice drink, UK | *Zygosaccharomyces bailii* | *Zygosaccharomyces parabailii* | SAMEA112469229 |
| 7850 |  |  | *Zygosaccharomyces bailii* | *Zygosaccharomyces pseudobailii* | SAMEA112469230 |
| 7851 |  | Sweet pickle | *Zygosaccharomyces bailii* | *Zygosaccharomyces pseudobailii* | SAMEA112469231 |
| 7852 | ATCC 8766,  CBS 749 | Sour red wine, USA | *Zygosaccharomyces bailii* | *Zygosaccharomyces bailii* | SAMEA112469232 |
| 7853 | ATCC 8099 |  | *Zygosaccharomyces bailii* | *Zygosaccharomyces parabailii* | SAMEA112469233 |
| 7854 |  |  | *Zygosaccharomyces bailii* | *Zygosaccharomyces parabailii* | SAMEA112469234 |
| 7855 |  |  | *Zygosaccharomyces bailii* | *Zygosaccharomyces parabailii* | SAMEA112469235 |
| 7856 |  |  | *Zygosaccharomyces bailii* | *Zygosaccharomyces parabailii* | SAMEA112469236 |
| 7857 | ATCC 56074,  CBS 2856 | Worcester sauce | *Zygosaccharomyces pseudobailii* | *Zygosaccharomyces pseudobailii* | SAMEA112469237 |
| 7858 | NRRL Y-7255 | Salad Cream | *Zygosaccharomyces bailii* | *Zygosaccharomyces parabailii* | SAMEA112469238 |
| 7859 | NRRL Y-7254 | Salad Cream | *Zygosaccharomyces bailii* | *Zygosaccharomyces parabailii* | SAMEA112469239 |
| 7860 | NRRL Y-7256 | Salad Cream | *Zygosaccharomyces bailii* | *Zygosaccharomyces parabailii* | SAMEA112469240 |
| 7862 | NRRL Y-11865 | Salad Cream | *Zygosaccharomyces bailii* | *Zygosaccharomyces parabailii* | SAMEA112469241 |
| 7863 |  | Anthocyanin Liquid from Grape skins | *Zygosaccharomyces bailii* | *Zygosaccharomyces parabailii* | SAMEA112469242 |
| 7864 | ATCC 10685,  CBS 684 | Honey | *Zygosaccharomyces bailii* | *Zygosaccharomyces pseudobailii* | SAMEA112469243 |
| 7865 | DBVPG 4426 | Wine or grape must | *Zygosaccharomyces bailii* | *Zygosaccharomyces bailii* | SAMEA112469244 |
| 7866 | DBVPG 4429 | Wine or grape must | *Zygosaccharomyces bailii* | *Zygosaccharomyces parabailii* | SAMEA112469245 |
| 7867 | DBVPG 4433 |  | *Zygosaccharomyces bailii* | *Zygosaccharomyces parabailii* | SAMEA112469246 |
| 7869 |  | Habitat: German kombucha tea, Germany | *Zygosaccharomyces bailii* | *Zygosaccharomyces bailii* | SAMEA112469247 |
| 7870 |  | Belgium | *Zygosaccharomyces bailii* | *Zygosaccharomyces parabailii* | SAMEA112469248 |
| 7871 |  | Ice Tea concentrate, Guatemala | *Zygosaccharomyces bailii* | *Zygosaccharomyces parabailii* | SAMEA112469249 |
| 7872 |  | Spoiled Dressing, Czech republic | *Zygosaccharomyces parabailii* | *Zygosaccharomyces parabailii* | SAMEA112469250 |
| 7873 |  | Yoghurt Dressing sachet, Czech republic | *Zygosaccharomyces parabailii* | *Zygosaccharomyces parabailii* | SAMEA112469251 |

^a^ all sequences submitted to the ENA under the study code PRJEB59101

^b^ as designated by Stratford et al (10)

^c^ as designated by Stratford et al (19)

^d^ as designated by Stratford et al (20)

**Table S2: Raw MIC*^EXP^* values used as input for inoculum effect screen of *Zygosaccharomyces* panel**

| Strain reference no. | MIC*^EXP^* (mM) for 10^2^ cells/well, biological replicate 1 | MIC*^EXP^* (mM) for 10^5^ cells/well, biological replicate 1 | MIC*^EXP^* (mM) for 10^2^ cells/well, biological replicate 2 | MIC*^EXP^* (mM) for 10^5^ cells/well, biological replicate 2 | MIC*^EXP^* (mM) for 10^2^ cells/well, biological replicate 3 | MIC*^EXP^* (mM) for 10^5^ cells/well, biological replicate 3 |
| --- | --- | --- | --- | --- | --- | --- |
| 1730 | 6 | 7.5 | 5 | 7 | * | * |
| 3697 | 5.5 | 11 | 6 | 10.5 | 5 | 6.5 |
| 3698 | 4.5 | 5.5 | 3.5 | 5.5 | 4 | 5 |
| 3699 | 5.5 | 7 | 5 | 6 | 4.5 | 5.5 |
| 3704 | 6 | 7 | 5.5 | 6.5 | 7 | 8.5 |
| 3942 | 5.5 | 7.5 | 6 | 8 | 5 | 8 |
| 3959 | 7 | 9 | 8 | 11 | 5 | 11 |
| 7406 | 7 | 8.5 | 4.5 | 7 | 7 | 9 |
| 7445 | 6 | 7.5 | 5.5 | 7 | 5.5 | 7 |
| 7769 | 6.5 | 8.5 | 5 | 8 | 5.5 | 7 |
| 7777 | 6.5 | 9 | 5 | 6 | 6 | 7.5 |
| 7788 | 6 | 7 | 5.5 | 7.5 | 5 | 6 |
| 7800 | 5 | 5.5 | 3 | 4.5 | 4 | 5.5 |
| 7807 | 6 | 7.5 | 4.5 | 6 | 5 | 6 |
| 7809 | 4.5 | 5.5 | 3.5 | 5.5 | 3.5 | 5.5 |
| 7812 | 5.5 | 6 | 5 | 7 | 5.5 | 6.5 |
| 7820 | 5.5 | 7 | 4 | 5.5 | 4.5 | 6.5 |
| 7829 | 6.5 | 7.5 | 3 | 3.5 | 5.5 | 7 |
| 7836 | 6 | 7.5 | 4 | 5.5 | 4.5 | 6 |
| 7838 | 6 | 6.5 | 5 | 6 | 3.5 | 4.5 |
| 7842 | 6.5 | 8.5 | 6.5 | 8 | 7 | 8.5 |
| 7843 | 5.5 | 6.5 | 4 | 5.5 | 5 | 9 |
| 7851 | 5.5 | 8.5 | 5 | 6 | 5 | 9 |
| 7852 | 4.5 | 5 | 3.5 | 4.5 | 3.5 | 4 |
| 7862 | 5.5 | 7.5 | 5 | 6.5 | 5.5 | 6.5 |
| 7870 | 7 | 8.5 | 7.5 | 9 | 6 | 6.5 |
| 7871 | 6.5 | 8.5 | 7 | 9.5 | 5.5 | 7 |
| 7873 | 6.5 | 9 | 4 | 5 | 6 | 8 |

*Contamination precluded measurement of third biological replicate

**Table S3: Wide panel of spoilage yeast isolates, including sorbic acid resistance values**

| **Species** | **Identifiers and isolation sources** | **Hetero-resistance (SD, mM)** | **IC_50_ (mM)** | **MIC*^MODEL^* (mM)** |
| --- | --- | --- | --- | --- |
| *Candida picinguabensis* | NY27, squeegee, soft drinks factory, UK | 0.0484 | 0.356 | 0.546 |
| *Rhodotorula mucilaginosa* | 95^a,b^, CMCC 2663, Vinyl wallpaper, UK | 0.0372 | 0.337 | 0.469 |
| *Rhodotorula glutinis* | 92^a,b^, Soft Drinks Factory, Israel | 0.0296 | 0.324 | 0.421 |
| *Trichosporon jirovecii* | 405^a,b^, Wooden pallet in de-palletizer, Turkey | 0.0246 | 0.684 | 0.850 |
| *Pichia membranifaciens* | 173^a,b^, UK | 0.0756 | 1.770 | 3.456 |
| *Zygosachharomyces parabailii* | NCYC 1766^a,b,c^, Blackcurrant and Grape juice | 0.0559 | 4.325 | 7.093 |
| *Zygosachharomyces bisporus* | NCYC 1555^b^, 134^a^, Salad cream | 0.0528 | 3.388 | 5.407 |
| *Cryptococcus laurentii* | 546^a,b^, Open drain, Sugar dissolving room, Brazil | 0.0239 | 0.638 | 0.789 |
| *Cryptococcus magnus* | 628^a,b^, scraper, factory, Russia | 0.0260 | 0.290 | 0.366 |
| *Candida boidinii* | 527^a,b^, Drain, Syrup room, Rio | 0.0520 | 1.694 | 1.774 |
| *Candida parapsilosis* | 69^a,b^, Tetrapak Fruit Juice, Baby formulation, UK | 0.0757 | 2.472 | 4.830 |
| *Wickerhamomyces anomalus* | 70^a^, NCYC 3371^b^,  Soft Drinks Factory, Israel | 0.0547 | 1.058 | 1.716 |
| *Kazachstania exigua* | 55^a,b^ CMCC 3199, Mayonnaise, UK | 0.0337 | 3.451 | 4.651 |
| *Torulaspora delbrueckii* | 529^a,b^, Under filler nozzle, Rio | 0.0236 | 2.307 | 2.842 |
| *Saccharomyces cerevisiae* | BY4741^a,b^ | 0.0240 | 2.477 | 3.064 |
| *Candida pseudointermedia* | 519^a,b^, Ingredient Room floor, Rio | 0.0464 | 0.958 | 1.444 |
| *Candida sojae* | 121^a,b^, Food factory, UK | 0.0447 | 1.897 | 2.817 |
| *Pichia kudriavzevii* | 522^a,b^, Syrup Room drain, Rio | 0.0447 | 3.258 | 4.839 |
| *Debaromyces hanseii* var *fabryii* | 100^a,b^, NCYC 3376, UK | 0.0101 | 1.535 | 1.678 |
| *Candida lusitaniae* | Collection of Malcolm Stratford, no previous designations | 0.0470 | 1.396 | 2.117 |
| *Zygotorulaspora florentina* | NCYC 2513^a,b^ | 0.0168 | 0.740 | 1.005 |
| *Zygosaccharomyces rouxii* | 597^a,b^, Dried Fruit, Spain | 0.0682 | 1.34 | 2.45 |
| *Zygosaccharomyces lentus* | 37^a^, NCYC 2789^b^,  Whole orange juice, Norwich, UK | 0.0437 | 6.22 | 9.16 |
| *Zygosaccharomyces pseudobailiii* | 2^a,b^, NCYC 563^c^, Sorghum Brandy | 0.0526 | 4.02 | 6.40 |
| *Zygosaccharomyces parabailii* | 9^a,b,c^, Carbonated orange soft drink, 10% fruit juice | 0.0881 | 4.01 | 8.74 |
| *Zygosaccharomyces parabailii* | 13^a,b,c^, USA | 0.946 | 4.11 | 9.50 |
| *Candida pseudolambica* | 525^a,b^, NCYC 3297,  Drain, Sugar dissolving room, Rio | 0.0465 | 3.27 | 4.94 |
| *Pichia manshurica* | 521^a,b^, Syrup Room drain, Rio | 0.0408 | 3.11 | 4.46 |
| *Saccharomyces cerevisiae* | 62^a^, NCYC 3368^b^,  CMCC 2268, Wine Yeast | 0.0360 | 3.64 | 5.00 |

^a^ as designated by Stratford et al (10)

^b^ as designated by Stratford et al (19)

^c^ as designated by Stratford et al (20)

**Table S4: Small panel of *Zygosaccharomyces* strains**

| **Reference** | **Species** | **Origin** |
| --- | --- | --- |
| 679 | *Z. parabailii* | NCYC 1766, Blackcurrant and Grape juice |
| 7810 | *Z. parabailii* | CFT-1, Canned tea Chicago |
| 7811 | *Z. parabailii* | CFT-10, Canned fruit, Chicago |
| 7812 | *Z. parabailii* | Phil-1, Bottled tea, Philadelphia |
| 7813 | *Z. bailii* | Preserved fruit punch |

**Supplementary Figures**


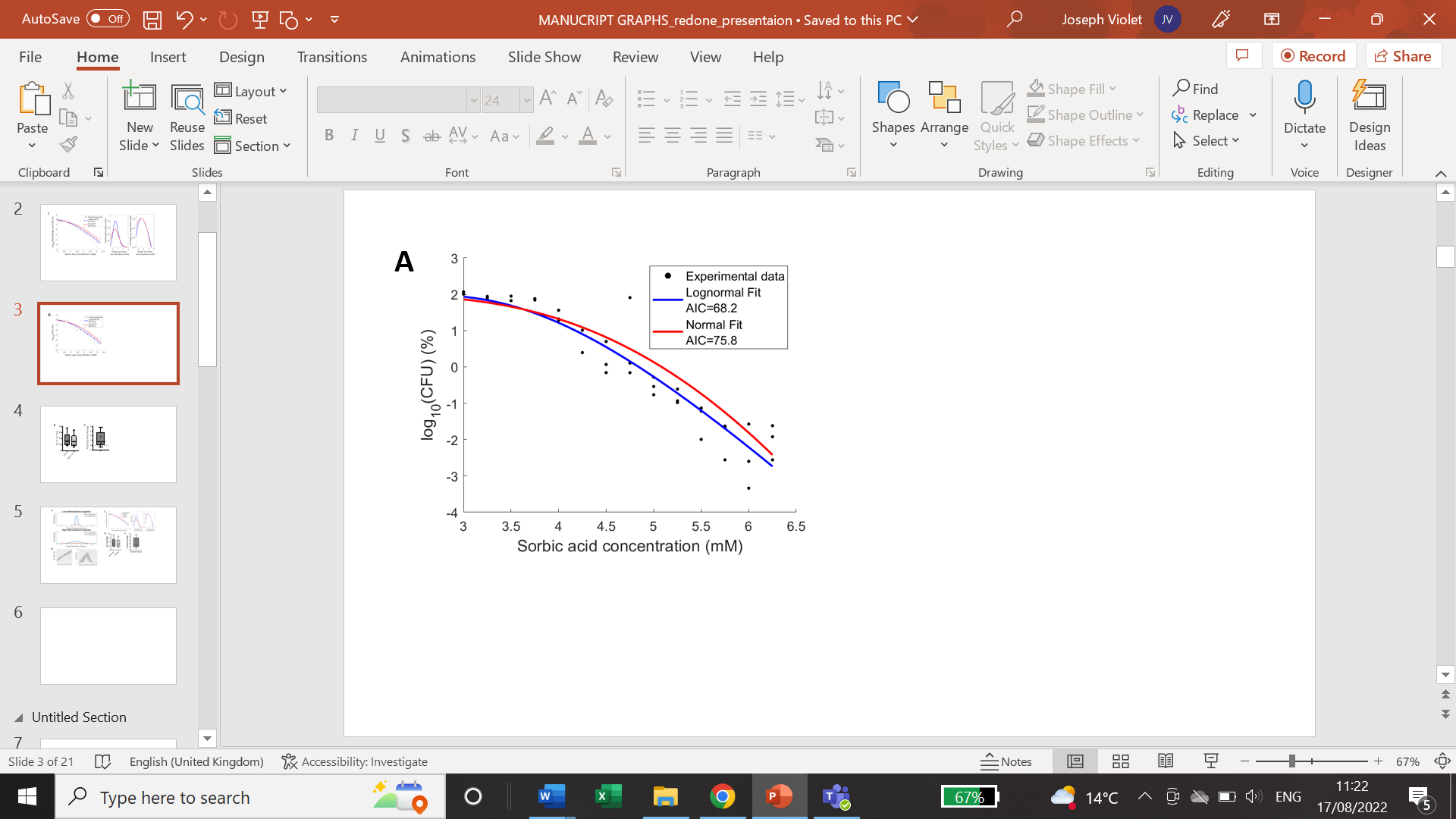

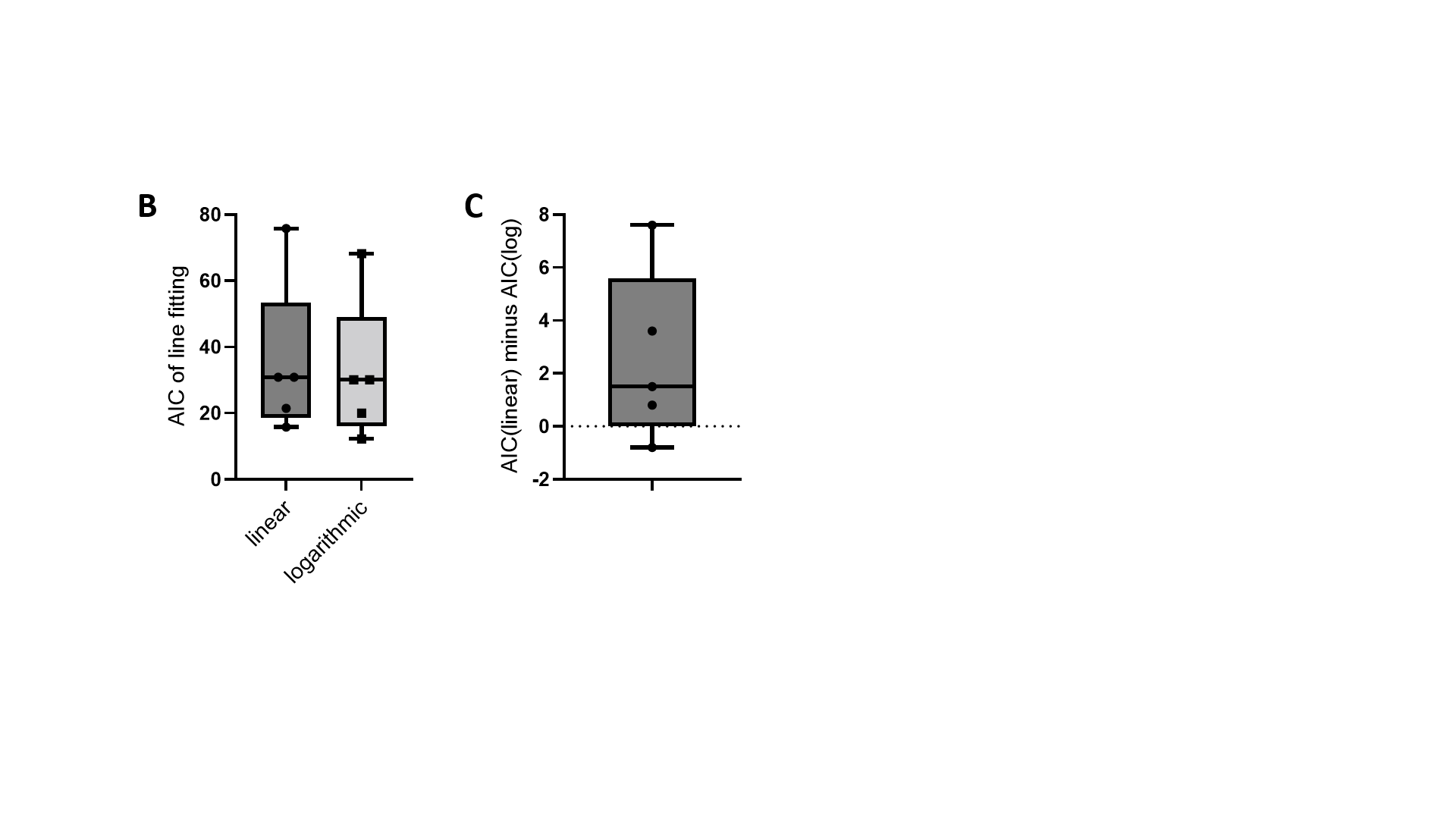


**Figure S1: Comparison of normal and lognormal distribution curves, for fitting of experimental dose-response curve data.**  **(A)** Example of dose-response curve, data for *Z. parabailii* 7812 on YPD agar supplemented as indicated with sorbic acid (n=3), with the experimental CFU (colony forming unit) data fitted to a lognormal (blue) or a normal (red) distribution. Modelling cell-cell resistances as normally distributed produces a heteroresistance value which relates to the *absolute* variation in single-cell resistances, whereas a lognormal distribution produces a heteroresistance value which relates to the *relative* variation in single-cell resistances. The result of this would be that a cell population with a relatively low IC_50_ would be given a relatively low heteroresistance value by a normal distribution curve fitting compared with that by a lognormal curve fitting, and the opposite would be true for a population with a high IC_50_. While a value reflecting relative variation in single-cell resistances is preferable, this analysis evaluates whether the different shape of the lognormal distribution curve could be accommodated by the experimental data. **(B)** Akaike Information Criterion (AIC) values for each curve fit are displayed (lower AIC indicates better fit, calculated in R), for dose-response curve data with sorbic acid for five *Zygosaccharomyces* strains (Table S2) fitted to normal and lognormal distributions. Points reflect curve fitting to three biological replicates for each sorbic acid concentration. **(C)** Pairwise difference in AIC between normal and lognormal fitting for dose-response curve data used in B, for each strain.

**Figure S2: Magnitude of cell density effect in *Z. parabailii* isolates with high heteroresistance (isolate 7445) and low heteroresistance (isolate 679).**

To discount the possibility that effects of inoculum cell density (e.g. due to stressor saturation or quorum sensing-type phenomena) rather than of total cell number, could contribute to MIC*^EXP^* increase between low and high inoculum size in the methodology, the effect of cell density on probability of growth occurring in a well was measured in high and low heteroresistance isolates. Each replicate value for cell density effect was calculated using growth probability (see Supplementary method) for *Z. parabailii* isolates 7445 and 679 at inoculum sizes of 10,000 cells/well (96 wells measured) and 100 cells/well (1920 wells measured). Experimental measurements of growth probability were determined after incubation of 96-well plates for 21 days at 23 °C. Growth probabilities were converted to Cell density effect using equations 1 and 2 (Supplementary method). Error bars are +/- SEM (n = 3 and 4 for isolates 7445 and 679, respectively). In these isolates, the 100-fold increase in cell concentration produces only a 15-17% increase in probability of *Z. parabailii* growth occurring in a well, with the remainder of the MIC*^EXP^* increase being attributable to the increased probability of a resistant cell being present at the higher cell-count (~100-fold). Cell concentration effects can be considered a minor component of the MIC*^EXP^* increase between inoculum sizes, relative to effect of likelihood that resistant cells occur in the inocula. The observation was consistent across strains characterised by high or low heteroresistance, with the small difference between cell concentration effect for these strains (15% and 17% increases in P, respectively) contrasting with a 51% difference in heteroresistance (0.0688 and 0.045 mM (SD) for strains 7445 and 679 respectively, according to the inoculum effect assay). Therefore, differences in MIC*^EXP^* between high and low inoculum sizes primarily reflect differences in heteroresistance rather than cell density effect, as per the assumptions of the inoculum effect assay.


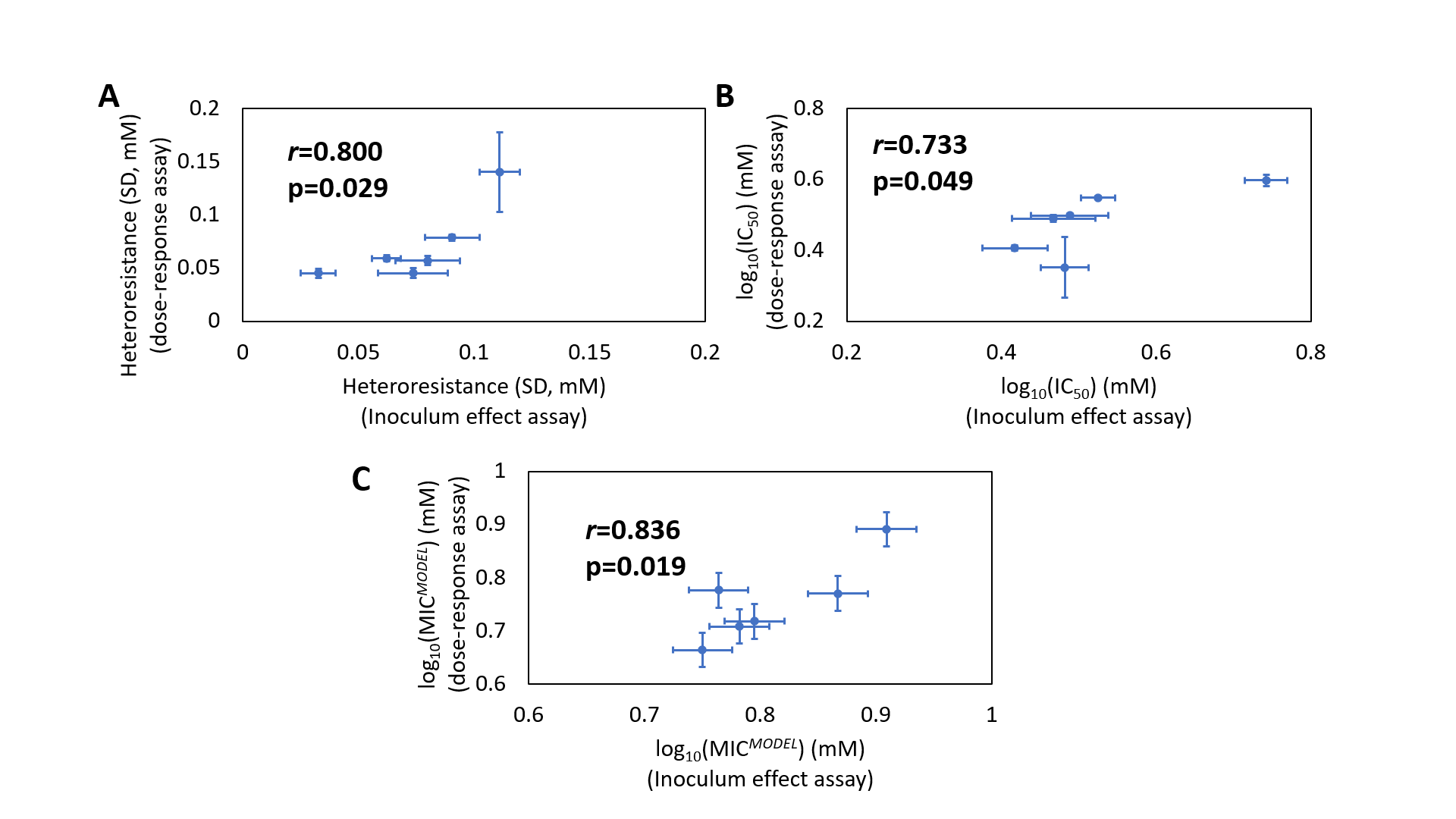


**Figure S3: Corroboration of inoculum effect assay on agar for measurement of IC_50,_ heteroresistance and MIC*^MODEL^*.**  Comparison of parameter determinations for randomly selected *Z. parabailii*, *Z. bailii* and *Z. pseudobailii* isolates (7788, 7812, 7851, 7829, 7769, 7842), selected to encompass a wide range of IC_50_, heteroresistance and MIC*^MODEL^* values. Parameter values for IC_50_ **(A)**, heteroresistance **(B)** and MIC*^MODEL^* **(C)** were derived either from dose-response curve on agar or inoculum effect assay on agar. A lognormal distribution model was used to fit curves to MIC*^EXP^* values obtained at two defined inoculum sizes (Inoculum effect assay method) or from percentage CFU counts at different sorbic acid concentrations (dose-response curve method). Points represent means from 6 and 3 biological replicates for dose-response and inoculum effect assays respectively for each isolate +/- SEM. Pearson’s r values were calculated using linear regression, p values were calculated using one-tailed linear regression.


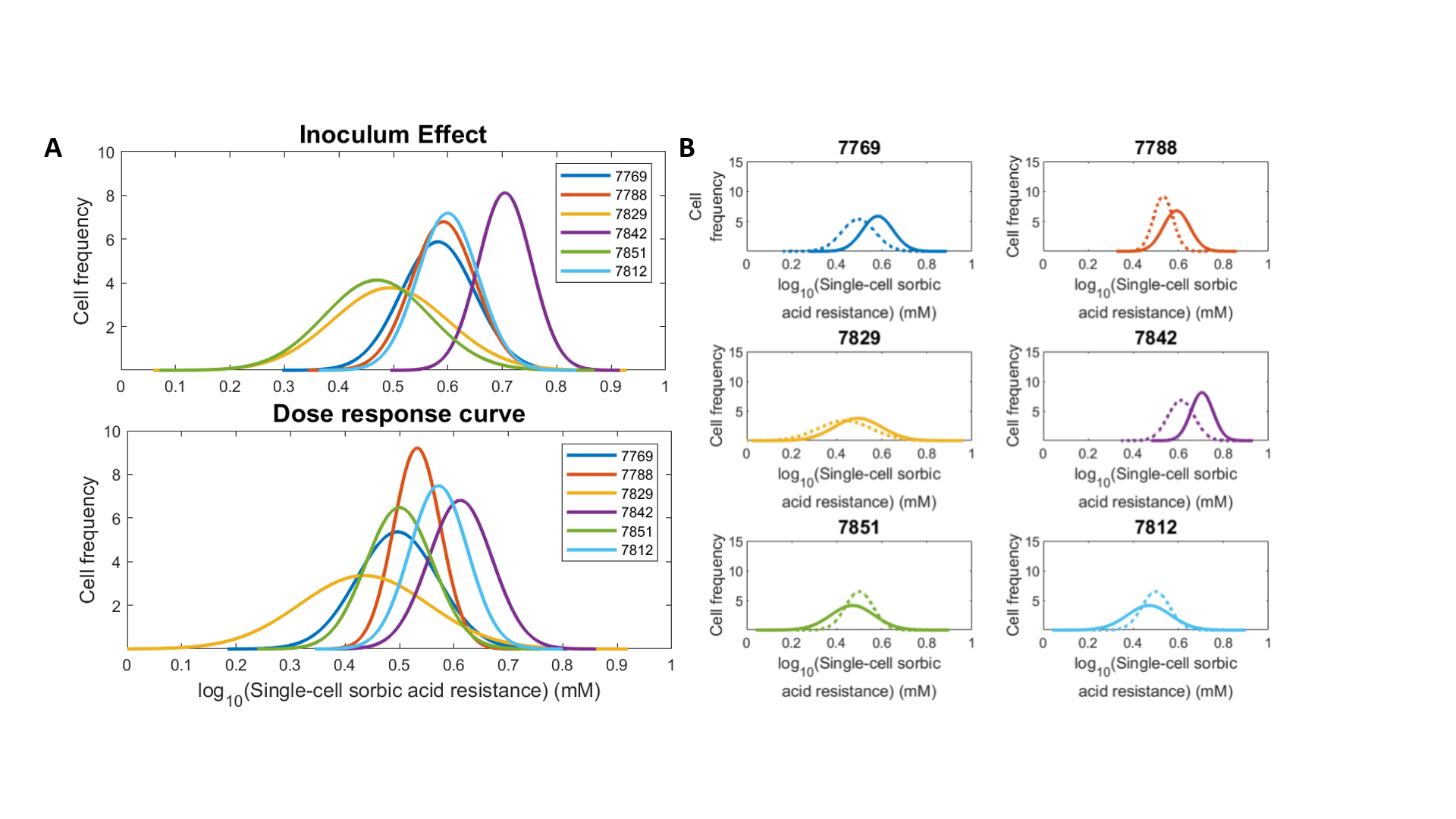


**Figure S4: Comparison of lognormal distribution curves of single-cell sorbic acid resistances predicted by inoculum effect methodology and dose response curves.** Selection of isolates (isolate numbers are shown) and predicted distribution curves were as in Figure 2. Distributions are displayed such that the total areas under curves = 1.0, and the y axis lower limit = 0.002, producing an x axis intersection approximating to MIC*^MODEL^*. **(A)** Lognormal distribution curves of single cell resistances predicted by inoculum effect (top) or dose response curve (bottom) for six isolates (n=6 biological replicates). **(B)** Curves shown in A, comparing inoculum effect (solid line) and dose response (dashed line) for each strain independently.


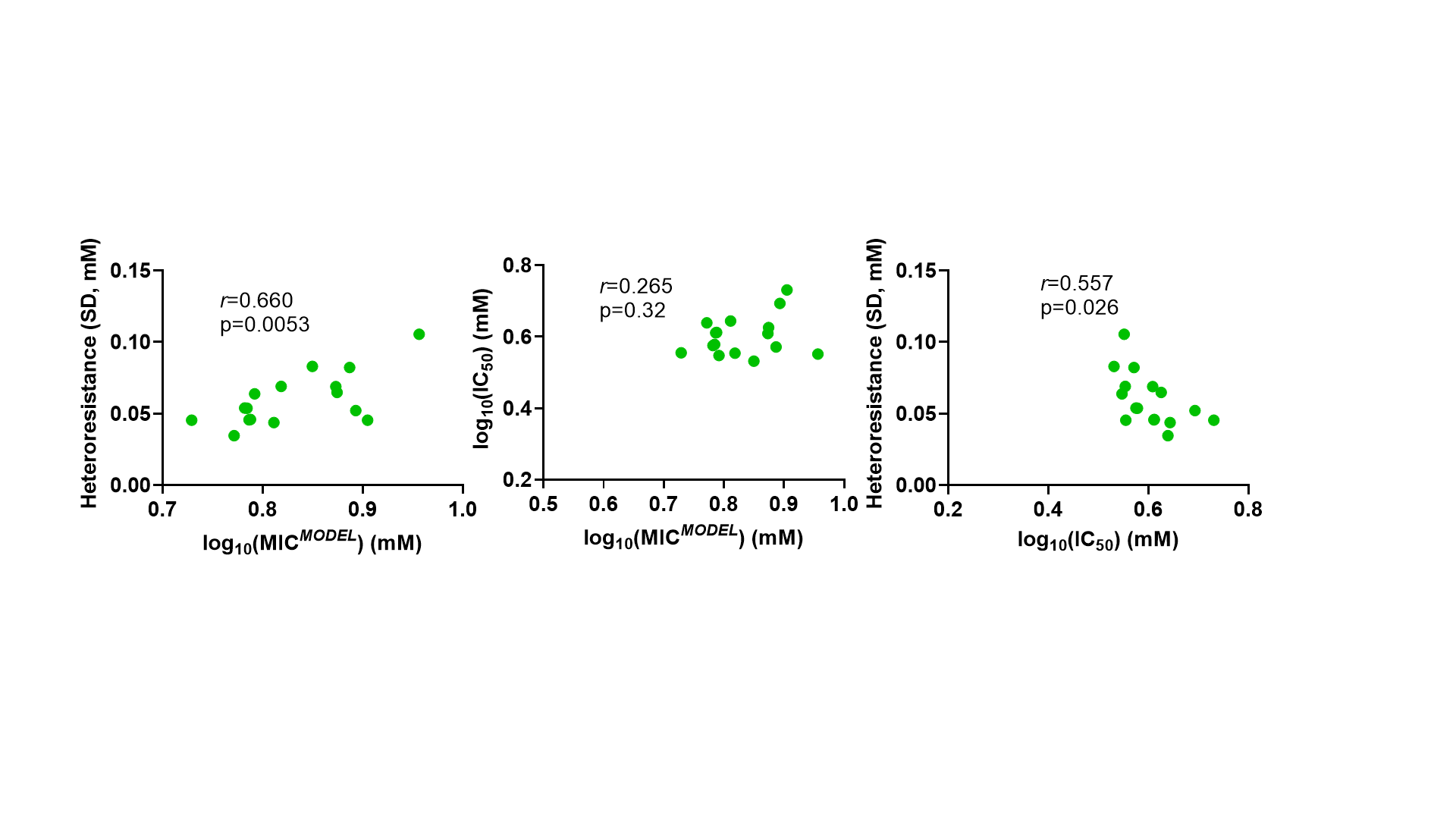


**Figure S5: Relationships between heteroresistance, IC_50_ and MIC*^MODEL^* in the *Z. parabailii* isolates of the *Zygosaccharomyces* sp. panel, omitting putative outlier isolate 3698.** Data were obtained as described in Figure 3. Pearson’s R and p values were produced from linear regression analysis (n=16).

**A**


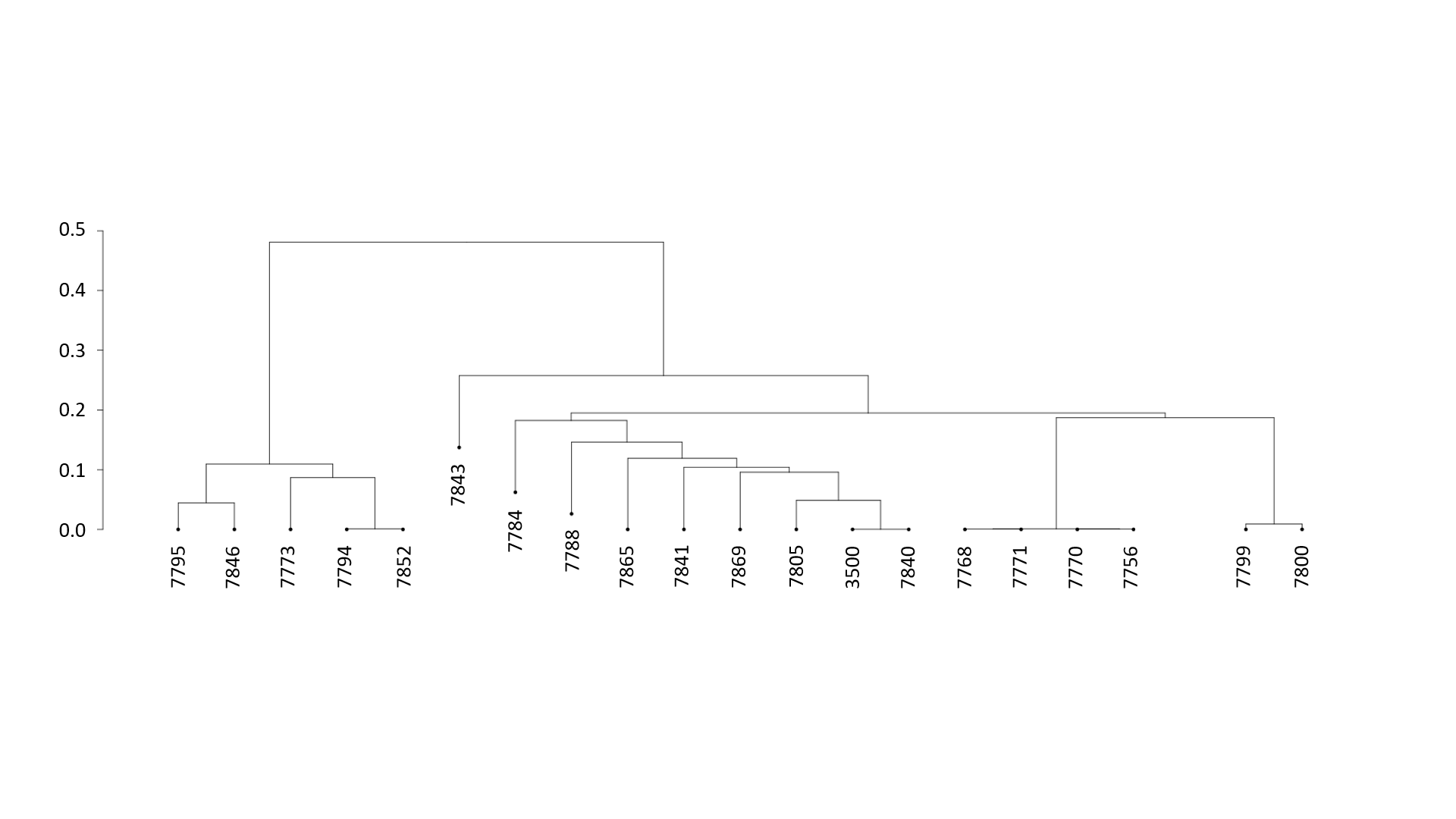


**B**


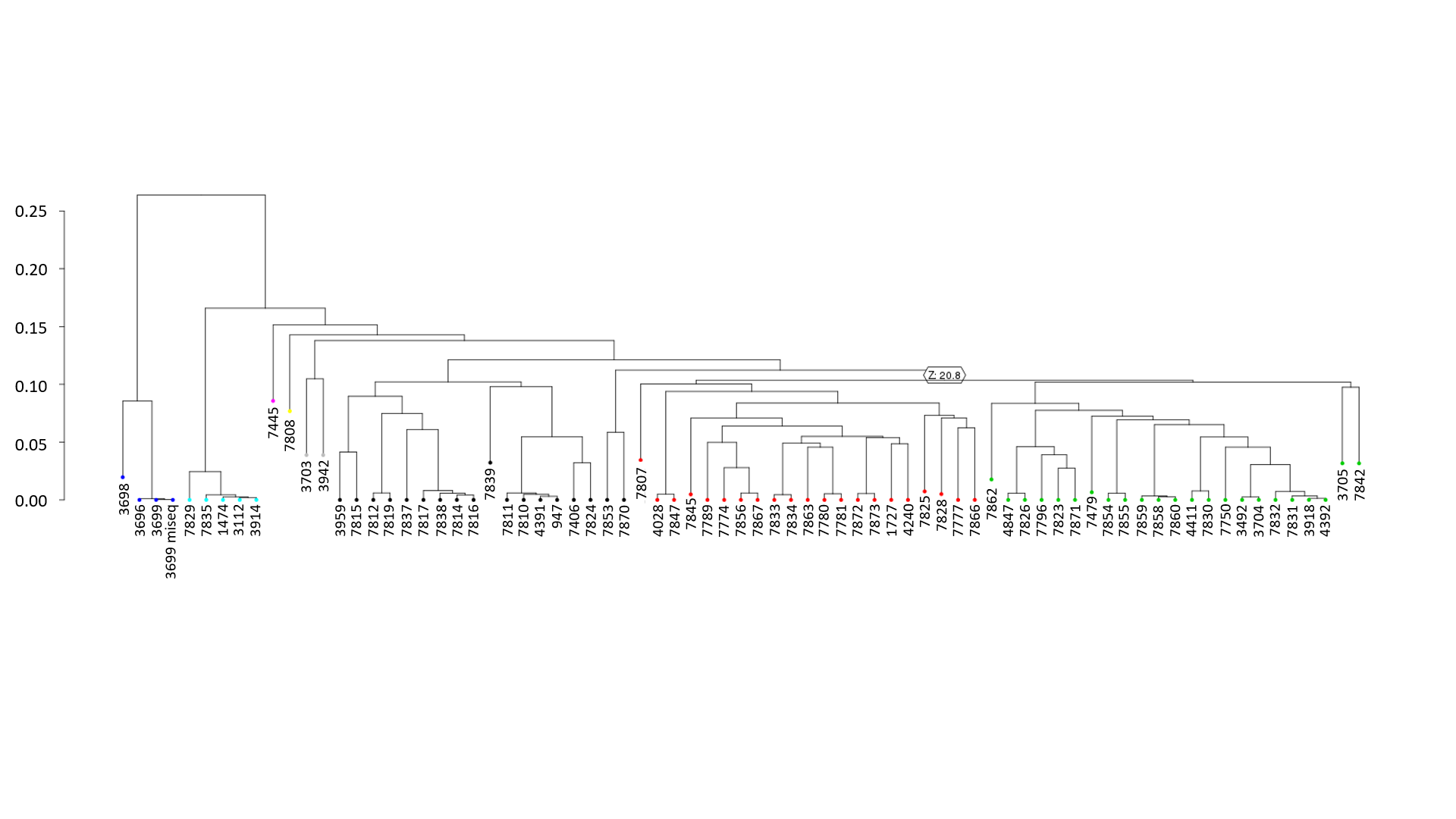


**C**


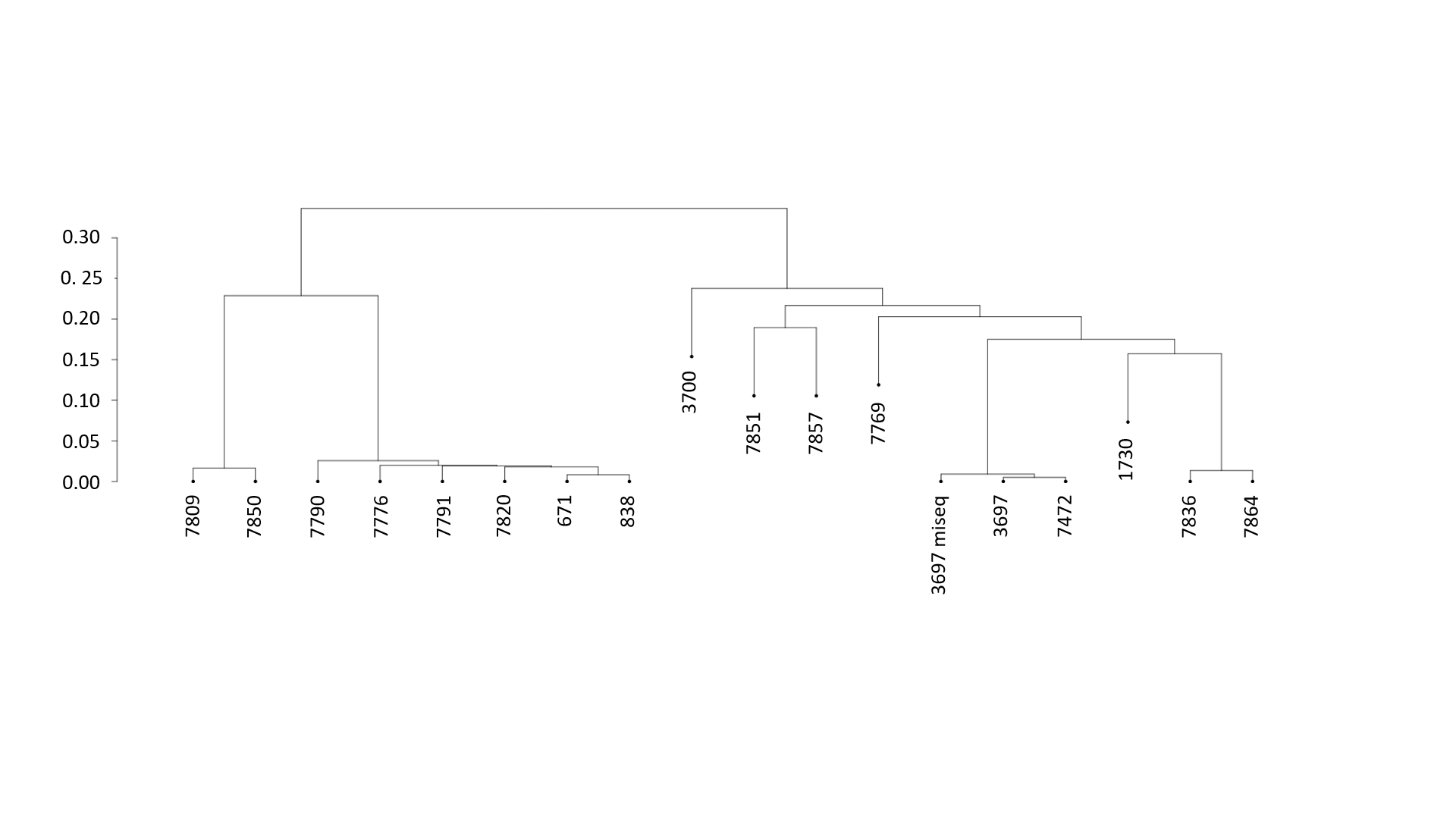


**Figure S6: Phylogenetic trees for isolates of *Z. bailii* (A), *Z. parabailii* (B) and *Z. pseudobailii* (C)**. *De novo* genome sequences were produced using PacBio^TM^ long reads and Illumina short reads for the reference strains marked ‘miseq’. Genome sequences for other isolates were produced by mapping Illumina short reads onto PacBio^TM^ long reads obtained for reference strains *(Z. bailii* 7846, *Z. parabailii* 3699, and *Z. pseudobailii* 3697). Relatedness values were produced using UPGMA scores from coding region alignments.
